# The EORTC emotional functioning computerized adaptive test: phases I–III of a cross-cultural item bank development

**DOI:** 10.1002/pon.3427

**Published:** 2013-11-11

**Authors:** Eva-Maria Gamper, Mogens Groenvold, Morten Aa Petersen, Teresa Young, Anna Costantini, Neil Aaronson, Johannes M Giesinger, Verena Meraner, Georg Kemmler, Bernhard Holzner

**Affiliations:** Department for Psychiatry and Psychotherapy, University Clinic for Biological PsychiatryAnichstraße 35, Innsbruck, Austria

## Abstract

**Background:**

The European Organisation for Research and Treatment of Cancer (EORTC) Quality of Life Group is currently developing computerized adaptive testing measures for the Quality of Life Questionnaire Core-30 (QLQ-C30) scales. The work presented here describes the development of an EORTC item bank for emotional functioning (EF), which is one of the core domains of the QLQ-C30.

**Methods:**

According to the EORTC guidelines on module development, the development of the EF item bank comprised four phases, of which the phases I–III are reported in the present paper.

Phase I involved defining the theoretical framework for the EF item bank and a literature search. Phase II included pre-defined item selection steps and a multi-stage expert review process. In phase III, feedback from cancer patients from different countries was obtained.

**Results:**

On the basis of literature search in phase I, a list of 1750 items was generated. These were reviewed and further developed in phase II with a focus on relevance, redundancy, clarity, and difficulty. The development and selection steps led to a preliminary list of 41 items. In phase III, patient interviews (*N* = 41; Austria, Denmark, Italy, and the UK) were conducted with the preliminary item list, resulting in some minor changes to item wording. The final list comprised 38 items.

**Discussion:**

The phases I–III of the developmental process have resulted in an EF item list that was well accepted by patients in several countries. The items will be subjected to larger-scale field testing in order to establish their psychometric characteristics and their fit to an item response theory model.

## Introduction

Assessment of health-related quality of life (HRQOL) is now a common practice in many cancer clinical trials, and emotional functioning (EF) is a common domain included in most HRQOL measures 1. The literature on screening instruments for distress in oncology patients is ever-expanding, and the implementation of routine screening in clinical practice is strongly suggested as a measure for improving cancer care 2 and embedded in the National Comprehensive Cancer Network distress management guidelines 3.

However, when applying traditional measurement approaches, there is always a trade-off between conciseness and precision. Screening instruments have only moderate sensitivities and specificities and may not be able to detect cancer patients in need of specialist intervention and support within cancer care 4,5. On the other hand, aiming at an accurate assessment usually requires a large number of questions, and many of these may lack relevance for the individual patient or a specific target population 4,6, resulting in considerable floor- and/or ceiling-effects.

Modern measurement approaches, such as item banking and computerized adaptive testing (CAT) 7, have great potential for overcoming some limitations of traditional assessment methods. Item banks enable the construction of questionnaire short forms designed for different purposes, such as a pre-defined level of measurement precision 10,11. With the help of computer technology, an item bank can also form the basis for CAT, an assessment method where a software program selects each question depending on the response to previous questions, guided by an underlying algorithm. CAT tailors the item set to the condition of each subject, thereby increasing measurement precision and the relevance of the instrument for the individual person. This is achieved without loss of comparability across subjects.

The European Organisation for Research and Treatment of Cancer (EORTC) Quality of Life Group (QLG) is currently working on the development of CAT versions 9,11–13 of the dimensions of its core questionnaire, the EORTC QLQ-C30 14. The intention is to generate a CAT version of the EORTC QLQ-C30 that can be used in lieu of the paper-and-pencil version, while maintaining comparability of results obtained from both versions. Hence, results obtained with the new CAT version can be compared with the substantial literature of studies using the QLQ-C30.

The EF is one of the core domains of the EORTC QLQ-C30. Previous studies have shown that there are special challenges in the development of a CAT instrument for the EF domain 16,17. First, there is no general definition of EF across different subject areas and key aspects vary across instruments. Second, the complex nature of the EF construct, with not only affective but also cognitive, behavioral, and somatic components, complicates its description within a common unidimensional item response theory (IRT) model. Within its large program of developing item banks for patient-reported outcomes, the Patient-Reported Outcome Measurement Information System (PROMIS) group has developed three distinct item banks for distress. Psychometric analyses included factor analytic and IRT methods and revealed a factor structure that led to the development of separate item banks for depression, anxiety, and anger. Each of these item banks comprises affective, cognitive, behavioral, and somatic aspects 17. The target populations for these measures are patients with a wide range of medical conditions. The psychometric approach to item bank development applied in this project is in line with the PROMIS approach, but the operationalized concepts differ in their scope. Whereas the PROMIS item banks are intended to address a number of different manifestations and levels of emotional distress, the EORTC EF-CAT is intended to mirror as closely as possible EF as conceptualized and assessed in the parent instrument, the QLQ-C30. In this paper, we describe the phases I–III of the development of the EORTC EF-CAT, that is, the development of an EF item list. This research is being conducted in an international context, where there is concern not only with psychometrics but also with cross-language and cross-cultural applicability. The latter are important in HRQOL assessment in general 18–20 and especially in the assessment of EF, as research suggests differences in the importance and in the reporting of emotions across cultures 21–23.

The specific aims of the research reported here included the following:

- determining an appropriate definition of EF as assessed by the EORTC QLQ-C30
- conducting a comprehensive literature search to identify items that might be appropriate for including in an EF item bank
- item selection and operationalization within a theoretical framework
- cross-cultural item pre-testing in cancer patients; and
- finalizing an EF item list for use in larger-scale, international field testing.

## Methods

Following the EORTC Module Development Guidelines, the EORTC item bank development project employs a multilingual and cross-cultural approach and includes a multi-level review process 24. The four development phases are (i) literature search, (ii) operationalization, (iii) pre-testing, and (iv) field testing 11. The current paper describes the phases I–III of the development of an EF item bank as well as the preceding conceptualization of the theoretical framework on which the item bank will be based.

The study has been approved by the ethics committees of the participating centers where patient interviews were conducted.

### Definition of theoretical framework and sub-domains

For the purpose of item bank and CAT development, the theoretical framework of EF as expressed in the parent instrument, the QLQ-C30 had to be refined. This included discussion on sub-domains (e.g., what is considered to be subsumed as general distress? and what about positive affect?), certain symptoms (e.g., the significance of suicidal thoughts) and considerations of how unidimensionality can be retained. Conceptualization was carried out in a focus group within the EORTC CAT group and discussed and finalized with senior members of the EORTC QLG.

#### Phase I: literature search and item collection

A literature search was carried out to obtain a broad picture of existing instruments and items measuring EF and of different operationalizations of EF. The retrieved list of EF items would serve as basis for our own item development. A combination of the following free text and MeSH-terms was used for the search: emotion*, well-being, function*, depression, anxiety, distress, questionnaire, instrument, scale, and assessment. These search terms were applied to MEDLINE, PSYNDEX, PSYNDEXplus Tests, PROQOLID, and the EORTC QLG item bank. The retrieved abstracts were examined for references to instruments/items that had been used to measure EF. All items listed as used to measure an aspect of EF was included at this stage. For all identified EF items, the item text, the instrument, and the response format were recorded.

#### Phase II: operationalization

The items identified via literature search were reviewed qualitatively by at least three reviewers concerning relevance to the EF concept as defined in phase I, redundancy, clarity, and appropriateness for the target population. Each reviewer received a template for each development step described later and rated the items independently. In case of disagreement, the items were discussed by the reviewers and a consensus choice was made. The results of each development step were evaluated by the project leaders and discussed if required.

The pre-defined development steps were the following:

1. Step 1: *Item classification according to theoretical framework*

Each item was classified to a sub-domain of EF on the basis of the developed theoretical framework (see results of phase I) or excluded if not fitting the concept.

1. Step 2: *Rating for redundancy and style*

Duplicates (i.e., similarly worded items) and items that were incompatible with the QLQ-C30 item style (which assesses severity/intensity on a Likert scale from ‘not at all’ to ‘very much’, with a reference frame of one week) were excluded.

1. Step 3: *Construction of items in QLQ-C30 style*

On the basis of the collected items, new items complying with the QLQ-C30 item style*s* were developed (i.e., original items were reformulated, split, etc.)

1. Step 4: *Rating of redundancy and relevance*

The items constructed in step 3 were again rated for redundancy and relevance. Redundancy in this selection step was defined by overlapping content of items.

1. Step 5: *Rating of item difficulty*

Items were classified into the categories ‘good EF’, ‘moderate EF’, or ‘poor EF’. ‘Good EF’ in this context refers to high levels of EF, that is, items that are mainly informative in patients with few or no distress. Conversely, items that are informative in patients with high levels of distress were classified as ‘poor EF’. An example of such a difficult item would be one that asked about feelings of hopelessness or uselessness. In case of insufficient coverage of the intended difficulty range, new items covering the under-represented areas of the EF continuum could be developed at this stage.

1. Step 6: *Cross-cultural expert reviews*

The items were evaluated for their relevance for the construct, appropriateness, and wording by (i) senior members of the QLG, (ii) methodological and clinical experts (psychometricians, physicians, and psycho-oncologists) within the CAT group, and (c) international expert panel. After each review, problematic items were discussed, and revised or deleted if needed.

#### Phase III: pre-testing

To assure content validity and the appropriateness of the items for the target population, the preliminary item list was subjected to pre-testing in cancer patients from different countries with different languages. The EORTC guidelines recommend a minimum of 10 patients per country 24. Translations were carried out by the Translation Office of the EORTC Quality of Life Department according to well-established guidelines 25. The pre-testing procedure comprised the administration of the item list and the QLQ-C30. A short structured interview to examine if items were difficult, confusing, annoying, or intrusive and to prompt the patient's description of additional significant issues was conducted by trained interviewers.

## Results

### Definition of theoretical framework and sub-domains

The QLQ-C30 EF scale comprises four items asking about feeling tense, worrying, feeling depressed, and being irritable (Table 1) assessing affective aspects of anxiety, depression, and general distress.

**Table 1:** Item list for field testing

Abbreviated item text	Sub-domain	Difficulty
Furious	GD	2
Anxious	A	2
Tense	A	2
Helpless	D	2
Panic	A	3
Lost interest in things (independently of actual ability)	D	2
Vulnerable	D	2
Frustrated	D	2
Worthless	D	3
Life meaningless	D	3
Angry	GD	2
Discouraged	D	2
Emotional outbursts	GD	3
Nothing could cheer you up	D	3
Afraid	A	2
Pleasure gone from life	D	3
Difficulty relaxing	A	2
Lost interest in your appearance	D	2
Restless	D	2
Miserable	D	2
Bad-tempered	GD	2
Depressed	D	3
Irritable	GD	2
Useless	D	3
Worry	A	2
Desperate	D	3
Difficult to stop or control worrying	A	3
Impatient	GD	2
Afraid of losing control	GD	2
Sad	D	2
Felt like giving up	D	3
Felt that nothing to look forward to	D	3
Felt that life isn't worthwhile	D	3
Carefree	PA	1
Satisfaction from things	PA	1
Calm	PA	1
Cheerful	PA	1
Felt inner strengths and abilities	PA	1

1 = good EF; 2 = moderate EF; 3 = poor EF.

GD, general distress; A, anxiety; D, depression; PA, positive affect.

The EF item bank should therefore cover the same affective domain and should not include somatic, behavioral, or cognitive aspects of EF. The affective manifestation of depression includes feelings of worthlessness, hopelessness, and sadness 26. The affective dimension of anxiety is characterized by nervousness, restlessness, and tension 26. Other negative emotional manifestations that may be considered symptoms of both anxiety and depression (e.g., anger or irritability), or that cannot be directly attributed to either of them (e.g., emotional outbursts), have been summarized as general distress.

With a focus on negative emotional aspects only, the best achievable state of EF would be the complete absence of any distress, with no regard for the presence of positive affect. Nevertheless, it was also decided to include positive emotional states within the same sub-dimensions to investigate their performance in patient interviews and psychometric analysis.

#### Phase I: literature search and item collection

The literature search was conducted in September 2008 and yielded 57 instruments (1729 items).

#### Phase II: operationalization

**Figure 1:**
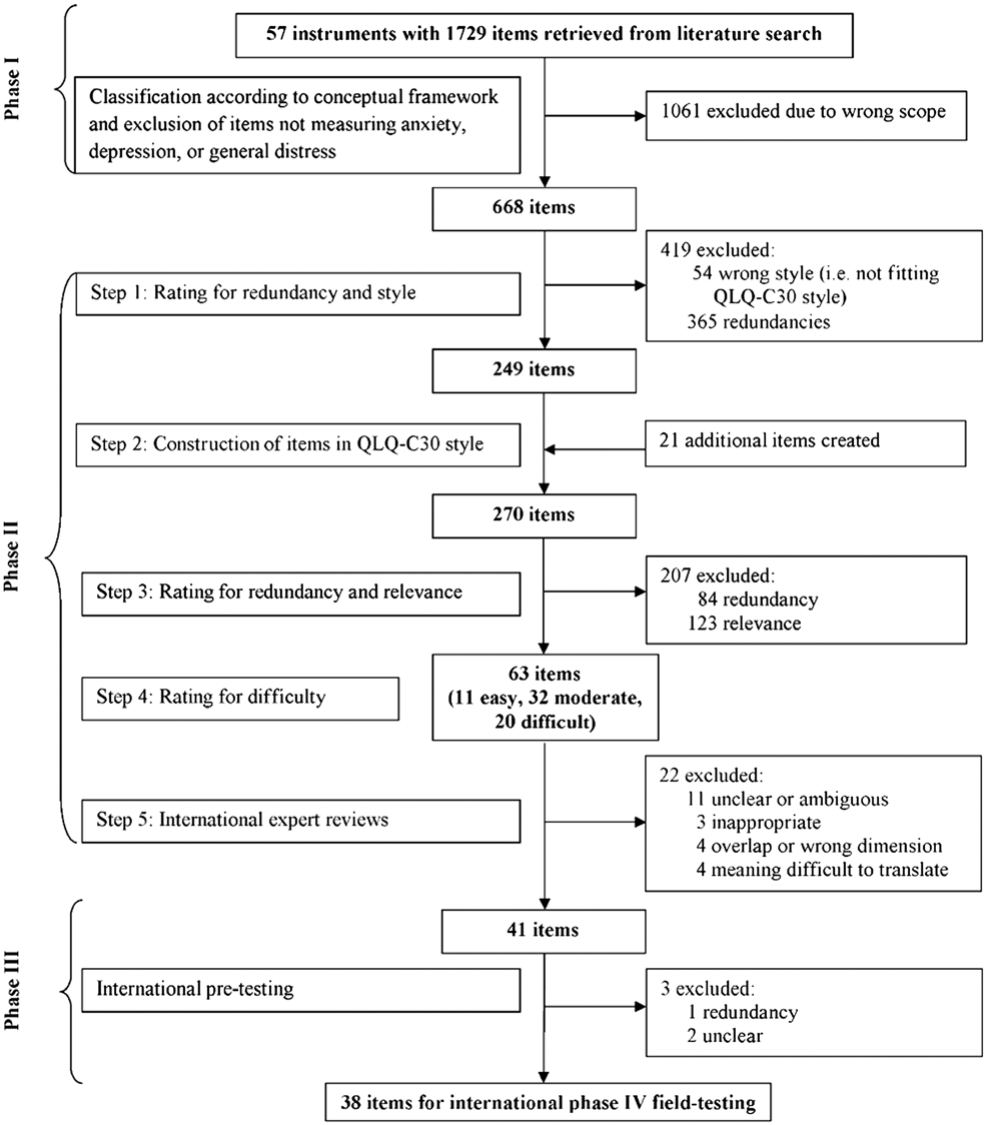
Flow diagram of item development process

On the basis of the items collected in phase I, pre-defined development steps were carried out. The details of this process, including numbers and reasons for item exclusions at each step, are outlined in the flow chart (Figure 1).

##### Steps 1–5

Altogether 1750 items were developed, reviewed, and selected. In total, 1238 items (71% of the original item collection) were excluded because of lack of relevance, incompatible style, or overlap with other constructs (e.g., ‘How much of your worries have you shared with any member of your family?’ was not considered to be relevant for the defined construct as not asking for severity/intensity). Because of the large number of items collected in phase I, redundancy rating was performed iteratively (step 2 and step 4) resulting in 449 (26% of the original item collection) exclusions. The resulting 63 candidate items were rated for difficulty: 17% were classified as ‘good EF’, 51% as ‘moderate EF’, and 32% as ‘poor EF’. This distribution was considered adequate for the target population. Across these development steps, between 13% and 31% of the items had to be discussed for consensus choice because of reviewer disagreements. Low agreement rates were observed primarily in redundancy ratings. This is not surprising given the large number of items initially evaluated.

##### Step 6

In step 6, the item list passed through a three-stage international review process in six different countries (Austria, Australia, Denmark, the Netherlands, Taiwan, and the UK), involving 14 experts on HRQOL and CAT. There was a clear consensus concerning the high relevance of the items for the EF item bank (85–100%). Changes that were prompted by the reviewers concerned the wording of nine items (e.g., ‘Have you felt that life isn't worth living?’ was changed to ‘Have you felt that life isn't worthwhile?’). Further, it was revealed that the items concerning death and dying required special attention. Finally, it was decided not to include such items because of the poor trade-off between gained information and their intrusiveness.

Overall, 22 items were deleted for being unclear or ambiguous, and inappropriate, or because of overlap with other constructs or measuring the wrong dimension. Four items were excluded as their meaning was difficult to translate from English into one or more other languages or would become redundant after translation (‘Have you felt downhearted?’, ‘Have you felt distressed?’, ‘Have you had crying spells?’, ‘Have you felt gloomy about things?’).

The resulting preliminary item list for cross-cultural patient interviews comprised 41 items.

#### Phase III: pre-testing

Patient interviews (*n* = 41) were conducted in four countries (Austria, Denmark, Italy, and the UK) in a sample of patients with a broad range of tumor types and stages (for details, see Table 2).

**Table 2:** Patient characteristics (*N* = 41)

Age (years)	Mean (SD)	63.5 (11.7)
	Range	29–82
Gender	Men	46.3% (19)
	Women	53.7% (22)
Nationality	Austria	24.3% (10)
	Denmark	24.3% (10)
	Italy	24.3% (10)
	United Kingdom	27.1% (11)
Marital status	Married/with partner	63.4% (26)
	No Partner	29.3% (12)
	Missing	7.3% (3)
Education (years)	0–10	41.5% (17)
	11–13	17.1% (7)
	14–16	17.1% (7)
	>16	21.9% (9)
	Missing	2.4% (1)
Employment	Full-time	26.8% (11)
	Part-time	4.9% (2)
	Unemployed	2.4% (1)
	Retired	58.5% (24)
	Other	4.9% (2)
	Missing	2.4% (1)
Tumor type	Anus	2.4% (1)
	Breast	19.5% (8)
	Colorectal and small intestine	26.9% (11)
	Gynecological	4.9% (2)
	Head and neck	9.8% (4)
	Kidney	2.4% (1)
	Lung	9.8% (4)
	Pancreatic	4.9% (2)
	Peritoneal	2.4% (1)
	Stomach	2.4% (1)
	Testicular	4.9% (2)
	Pleura mesothelioma	2.4% (1)
	Pulmonalsynovialsarkoma	2.4% (1)
	Missing	4.9% (2)
Stage	I–II	31.7% (13)
	III–IV	56.1% (23)
	Missing	12.2% (5)
Current treatment	No	34.1% (14)
	Chemotherapy	58.5% (24)
	Radiotherapy	2.4% (1)
	Radio chemotherapy	2.4% (1)

After excluding overall comments referring to the similarity of items or concerning the response format, 28 items were commented upon by at least two patients. Fifteen items were considered confusing and 19 difficult to understand/answer by one or more (maximum of four) patients. Hardly, any items were rated annoying, upsetting, or intrusive. Concerning the items on positive affect some explicitly supported asking this kind of questions (e.g., ‘More positive questions. It is very depressing to read/answer all the negative questions.’), whereas others found them confusing. When in doubt about the exclusion of an item, we tended to keep it, so it can be evaluated on the basis of the significantly larger sample of patients to be recruited in field testing.

On the basis of patient feedback, the item ‘Have you lost interest in things, such as recreational or social activities?’ was complemented with ‘(independently of your actual ability to do them)’. Furthermore, ‘Did you feel tense?’ and ‘Have you felt nervous?’ were considered similar on the basis of patient feedback, and thus, the original QLQ-C30 item ‘Did you feel tense?’ was retained. The items ‘Have you had pessimistic thoughts?’ and ‘Have you had frightening thoughts or feelings?’ were deleted for being unclear.

The patients in our sample had rather high levels of EF (72% had an EF score above the median of 75 reported for cancer patients in general in the QLQ-C30 reference manual 27). In addition, the majority of items rated difficult were those addressing relatively severe levels of depressive states (such as feeling worthless). Thus, one reason that this type of items were perceived difficult to answer for some patients might be that currently, they could not relate to the high level of emotional distress involved. This problem would be avoided within a CAT procedure.

After these development steps, the provisional item list consisted of 38 items (Table 1; 5 judged to assess good EF, 20 moderate EF, and 13 poor EF). Content domains where covered as follows: 7 items assessed aspects of anxiety, 19 items depression, 7 items general distress, and 5 items positive affect.

## Discussion

The present article describes phases I–III of the development of the EORTC EF-CAT and gives details on the conceptual considerations and development steps that were followed. The concept of EF applied in item bank development adheres to the conceptualization of EF as measured with the EORTC QLQ-C30, that is, defined as a single affective domain, and all EORTC CAT versions are designed to mirror the conceptualization and content coverage of the original version of the questionnaire. The development process was based on the EORTC guidelines for module development 24, adapted to CAT development 11 and corresponds to commonly suggested guidelines for item bank development 7,28. The resulting item list comprised 38 items and will be subjected to rigorous psychometric evaluations in phase IV to shape the final item bank and to calibrate the items for the use in the EORTC EF-CAT.

As with all EORTC QOL instruments, the new item bank needed to be designed to meet the requirements of an instrument that is administered to cancer patients in a range of countries and cultural settings. Thus, the principal foci in the development of the item list were the elaboration of the definition of EF to be operationalized in the new item bank, the appropriateness of the questions for the target population, and the cross-cultural applicability.

One major advantage of the approach applied in this study is not only the early incorporation of feedback from experts' in the fields but also the patients from different cultural settings, which is important for improving content validity 7,28. A problem that arose in the international patient testing was that only a few patients with moderate to severe EF impairments were interviewed (EF scores >75 in 72%). Unlike in the final CAT version, all patients in the testing phase are required to answer all questions, so patients with impaired EF could be burdened by the large number of questions. There was, however, no indication that patients with impaired EF had more difficulties with the items. Overall, the items were perceived relevant and appropriate, and the items were improved in terms of wording and scope within this review process.

The cross-cultural approach as well as considerations of dimensionality distinguishes the EORTC EF-CAT item bank from item banks on emotional distress developed by the PROMIS group in the USA. PROMIS is the largest initiative on item bank and CAT development worldwide. Their measures are initially developed for a broad but English-speaking target population. Most of them have been translated into Spanish, and translations into other languages are ongoing 32. For the assessment of emotional distress they have, on the basis of conceptual and psychometric grounds, decided on a multidimensional approach and thus developed three distinct item banks on depression, anxiety, and anger, respectively. All three item banks comprise affective, cognitive, behavioral, and somatic symptoms. The PROMIS item banks facilitate a comprehensive assessment of emotional distress.

As mentioned previously, within the EORTC CAT-project, EF had to be operationalized in such a way as to maintain comparability with the original QLQ-C30 EF domain. Thus, we aimed at a unidimensional description of the construct, incorporating aspects of anxiety, depression, and general distress but including affective symptoms only. It is important to note that the CAT-EF is not intended to reflect a psychiatric diagnosis or disorder. In the context of somatic illness, commonly used diagnostic criteria for psychiatric disorders often are not useful descriptors for patients' emotional distress 33–35. Furthermore, in cancer patient populations, it can be difficult to distinguish between symptoms that reflect impairments in EF rather than somatic health problems (e.g., fatigue or appetite loss caused by the disease or treatment). Thus, although emotional distress can manifest itself in various ways, focusing on the affective aspects seems sensible for the purpose of the item bank presented here and has several advantages. First, our development approach might facilitate building a unidimensional model for EF, which is a crucial issue for standard IRT calibration. Second, the present approach avoids confounding cancer-related and treatment-related somatic symptoms and somatisation, as well as overlap with other domains measured by the QLQ-C30 such as social, role, and cognitive functioning. Third, from a practical point of view, having one outcome parameter for EF will simplify interpretation of results not only in a research context but also in clinical practice. And finally, because of adhering to the concept of EF as measured with the QLQ-C30, results based on the EF item bank can be compared with data collected in the many studies that have used the original QLQ-C30. Although the EF item bank cannot provide a detailed assessment of emotional distress in its various manifestations, it does provide an estimate of the overall level of emotional distress.

In phase IV, the provisional list of 38 items will be field-tested in a sample of 1000 cancer patients from four countries (Austria, Denmark, Italy, and the UK). Analyses will include factor analytic methods to investigate dimensionality, IRT analyses to calibrate the item bank, and investigations of differential item functioning. These psychometric analyses will indicate whether the EF items fit a unidimensional model while maintaining content balance and will form the basis for the final selection and calibration of items for the item bank. In developing the model for the final item bank, item selection will, however, not be based on psychometric analysis only but will also take into consideration the clinical importance of items 9.

In summary, the EORTC EF item bank and EF-CAT will provide a range of advantages in the assessment of the emotional dimension of HRQOL in the oncology context. The EORTC EF item bank will facilitate the construction of different short forms while maintaining comparability of results. Considering that 25–40% of cancer patients present with heightened levels of psychosocial distress that requires professional intervention 31–33, this appears especially advantageous for screening purposes, as such a short-form can be designed to have maximum precision at the cut-off for relevant distress.

The CAT facilitates keeping the assessment not only short but also relevant for the individual. This may, for example, play an important role in home-monitoring programs, where frequent assessments with narrow intervals 35 make assessment methods prone to increased patient burden and missing data. Last but not the least, the algorithm that is used to guide the selection of items can be designed to ensure content coverage, that is, ensure that in each assessment, items from each sub-dimension (anxiety, depression, and anger) are used. Compared with instruments based on classical test theory, the EORTC EF-CAT will facilitate covering a range of affective symptoms with a minimum of questions while minimizing measurement error at the individual and group level.
